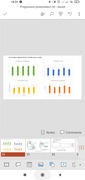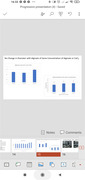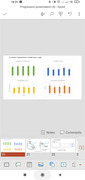# Physical Characterisation of pre‐clinical progressive model of Alzheimer’s Disease

**DOI:** 10.1002/alz.087945

**Published:** 2025-01-03

**Authors:** Abdullah Asad Iqbal, Bushra Almari

**Affiliations:** ^1^ University of Huddersfield, Huddersfield, Yorkshire United Kingdom

## Abstract

**Background:**

Alzheimer’s Disease research lacks a suitable model to match the sporadic version of Alzheimer’s Disease (SAD). We a propose a model that will use 7PA2 cells which is a CHO modified to express the V717F mutation for APP (Indiana mutation). The 7PA2 cells will then be placed inside alginate microbeads to produce a factory that constantly produces amyloid species. Then the injected into the brains of rats. Generating pathological changes which can be compared against previous models and human data to test whether this model is more appropriate than current models.

**Method:**

Alginate beads where produced of various different concentrations of alginate (Alg) and the gelling agent (calcium chloride ‐ CaCl2). 0.4 ‐ 8%Alg and 0.1M CaCl2 ‐ 1M CaCl2.

Stability measured as change in diameter using a Keynes microscope and comparing diameter for beads containing bv2 cells and no cells.

Stability also compared in aCSF.

Young Modulus was measured using a texture analyser and rheometer.

Inflammatory effects of alginate on BV‐2 cells characterised through TNF‐alpha.

**Result:**

We found no changes in stability with changes in Alg or CaCl2 or if beads were in aCSF or distilled water for 4 weeks. (n = 50).

No significant effects of alginate on TNF‐alpha expression levels.

Young Modulus of Alg beads of 1.5% Alg and 0.3%M closely matches brain environment.

**Conclusion:**

Alginate beads are stable over time in a brain‐like environment. Furthermore alginate beads of 1.5% Alg and 0.3%M closely match the brain environment. Alginate also causes no significant inflammatory response. Therefore alginate is a suitable carrier for 7PA2 further work must now be done in characterising 7PA2 cells and their amyloid species and the eventual effects in the brain.